# MSDAFL: molecular substructure-based dual attention feature learning framework for predicting drug–drug interactions

**DOI:** 10.1093/bioinformatics/btae596

**Published:** 2024-10-09

**Authors:** Chao Hou, Guihua Duan, Cheng Yan

**Affiliations:** School of Informatics, Hunan University of Chinese Medicine, Changsha, Hunan 410208, China; School of Computer Science and Engineering, Central South University, Changsha, Hunan 410083, China; School of Informatics, Hunan University of Chinese Medicine, Changsha, Hunan 410208, China

## Abstract

**Motivation:**

Drug–drug interactions (DDIs) can cause unexpected adverse drug reactions, affecting treatment efficacy and patient safety. The need for computational methods to predict DDIs has been growing due to the necessity of identifying potential risks associated with drug combinations in advance. Although several deep learning methods have been recently proposed to predict DDIs, many overlook feature learning based on interactions between the substructures of drug pairs.

**Results:**

In this work, we introduce a molecular Substructure-based Dual Attention Feature Learning framework (MSDAFL), designed to fully utilize the information between substructures of drug pairs to enhance the performance of DDI prediction. We employ a self-attention module to obtain a set number of self-attention vectors, which are associated with various substructural patterns of the drug molecule itself, while also extracting interaction vectors representing inter-substructure interactions between drugs through an interactive attention module. Subsequently, an interaction module based on cosine similarity is used to further capture the interactive characteristics between the self-attention vectors of drug pairs. We also perform normalization after the interaction feature extraction to mitigate overfitting. After applying three-fold cross-validation, the MSDAFL model achieved average precision scores of 0.9707, 0.9991, and 0.9987, and area under the receiver operating characteristic curve scores of 0.9874, 0.9934, and 0.9974 on three datasets, respectively. In addition, the experiment results of five-fold cross-validation and cross-datum study also indicate that MSDAFL performs well in predicting DDIs.

**Availability and implementation:**

Data and source codes are available at https://github.com/27167199/MSDAFL.

## 1 Introduction

Drug–drug interactions (DDIs) can cause unexpected adverse drug reactions, affecting treatment efficacy and patient safety (Vilar *et al.* 2014). DDIs refer to interactions that occur between two or more drug administration processes, including changes in drug properties and the occurrence of toxic side effects (Sun *et al.* 2016). Therefore, research on DDI prediction is of great practical importance. However, traditional biological or pharmacological methods are costly, time-consuming, and labor-intensive (Shao and Zhang 2013).

Machine learning offers a fresh avenue for accurately predicting DDIs (Mei and Zhang 2021). Methods based on feature similarity posit that drugs sharing similar attributes often exhibit comparable reaction patterns, relying largely on drug properties such as fingerprinting (Vilar *et al.* 2013), chemical structures (Takeda *et al.* 2017), pharmacological phenotypes (Li *et al.* 2015), and RNA profiles (Li *et al.* 2022). Enhancements in model efficacy are achieved by integrating various features. For instance, the DDI-IS-SL model forecasts DDIs through a blend of integrated similarity measures and semi-supervised learning techniques (Yan *et al.* 2020). Despite their advancements, these feature similarity-based methods often overlook the structural details of drugs, and their feature selection heavily depends on specialized knowledge and experience.

Graph neural networks (GNNs) have widely been implemented to analyze the chemical structures of drugs and forecast DDIs. Contemporary GNN methodologies are divided into two main types. The first type focuses on embedding features directly from the molecular graphs of drugs, effectively utilizing a straightforward method to encapsulate graph-based data (Gilmer *et al.* 2017). In this method, atoms within the molecular graph are treated as nodes, with chemical bonds serving as the connecting edges. This setup allows for the embedding of the molecular graph by learning features of individual atoms and the interactions conveyed through the chemical bonds. For instance, SSI-DDI deconstructs the DDI prediction task between two drugs to pinpoint pairwise interactions among their respective substructures (Nyamabo *et al.* 2021). DSN-DDI is a dual-view drug representation learning network specifically engineered to concurrently learn drug substructures from individual drugs and drug pairs (Li *et al.* 2023). The second type leverages existing drug interaction networks, where drugs are nodes and their interactions are edges, treating the task of DDI prediction as akin to link prediction within these networks. Clearly, the latter type exhibits a common limitation: they lack inductive capability, are unable to accommodate novel drugs not present in the interaction network, and struggle to maintain diverse types of associations between entities. KGNN applies a knowledge-based GNN approach to extract relational data from knowledge graphs to enhance DDI prediction (Lin *et al.* 2020). MIRACLE employs multi-view graph contrastive representation learning to simultaneously capture the structural interplay and interactions within and between molecules, enhancing the prediction of DDIs (Wang *et al.* 2021). Lastly, HTCL-DDI applies a hierarchical triple-view contrastive learning framework for predicting DDIs (Zhang *et al.* 2023). These diverse approaches illustrate the adaptive use of GNNs in addressing the complexities of predicting drug interactions, combining structural and relational data for improved predictive accuracy.

While current deep learning approaches have demonstrated promising results in predicting DDIs, there remains considerable potential for further enhancement. Firstly, methods that rely solely on a single self-attention mechanism for feature extraction may not comprehensively characterize drug information, potentially missing complex interactions between different substructures. Additionally, integrating multiple sources of feature information could introduce redundant features and noise, unnecessarily complicating the model. Secondly, overfitting during model training significantly impacts prediction results, often resulting in biased predictions. The main contributions of this work are outlined as follows:

- We designed a new Molecular Substructure-based Dual Attention Feature Learning framework for predicting DDIs (MSDAFL). This framework leverages both self-attention and interactive attention mechanisms to effectively extract and process interaction information between drug substructures, enhancing the accuracy of DDI predictions.
- To uncover the hidden features of interactions between drug substructures, we computed the cosine similarity matrix. This approach has shown that these similarity vectors significantly contribute to the accuracy of predicting DDIs.
- Additionally, to reduce overfitting during model training, we adopted a normalization strategy. This not only retains the essential interaction features but also improves the predictability and reliability of DDI outcomes.

## 2 Materials and methods

### 2.1 Dataset

To evaluate the scalability and robustness of MSDAFL, we test our model on three public datasets, which vary in scale, density and widely used in previous studies. The scale of the dataset is determined by the number of drugs included. According to previous studies, we also treat the observed DDIs as positive samples and also randomly sample the non-existing DDIs to generate the negative samples. We perform stratified splitting to divide all the drug pairs into a training set, a validation set, and a testing set in a ratio of 6:2:2 (three-fold cross-validation) and 8:1:1 (five-fold cross-validation). We run the experiments on three random folds and five random folds, respectively. As shown in Supplementary Table S1, the statistics of the preprocessed datasets are listed as follows:

- ZhangDDI dataset (Zhang *et al.* 2017) is of small-scale, consisting of 544 drugs and 45 720 pairwise DDIs.
- ChCh-Miner dataset (Ma *et al.* 2018) is of medium-scale, consisting of 997 drugs and 21 486 pairwise DDIs.
- DeepDDI dataset (Gilmer *et al.* 2017) is of large-scale, consisting of 1704 drugs and 191 870 pairwise DDIs.

### 2.2 Problem formulation

The DDI prediction task is formulated as a binary classification problem aimed at discerning the existence of interactions between pairs of drugs. A drug’s molecular structure can be abstractly represented by a graph *G*, where $X\in R^{N\times d}$ denotes the node feature matrix and $A\in R^{N\times N}$ denotes the adjacency matrix. Within molecular graphs, nodes correspond to atoms, and edges represent chemical bonds between them. GNNs primarily employ the Message Passing mechanism, which integrates information from neighboring nodes to update their representations (Gilmer *et al.* 2017). Prominent variants of GNNs include Graph Convolutional Networks (GCNs) (Kipf and Welling 2016), Graph Attention Networks (GATs) (Velickovic *et al.* 2017), and Graph Isomorphism Networks (GINs) (Xu *et al.* 2018). In this study, we adopt GIN as the foundational architecture for our model. The detailed description of how construct the node feature matrix **X** is illustrated in Supplementary Section S2. In the context of DDI prediction, given the adjacency matrix **A** representing the molecular structure graph *G* and the node feature matrix **X**, the objective is to derive a predictive function $f(d_{1},d_{2})\to [0,1]$. This function aims to estimate the likelihood of interaction between any pair of drugs $d_{1}$ and $d_{2}$.

### 2.3 Overview of MSDAFL

**Figure 1.**
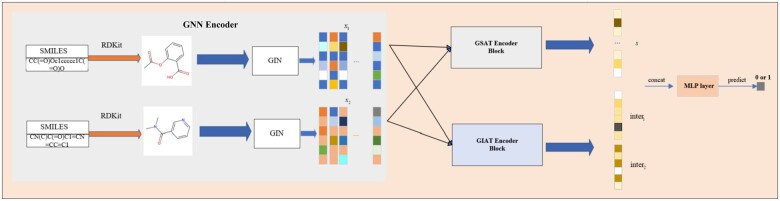
Overview of the proposed MSADAFL framework. The overall framework includes a GNN encoder, a self-attention mechanism encoder (GSAT encoder), and an interactive attention mechanism encoder (GIAT). The features from GSAT and GIAT are concatenated in an MLP for prediction, yielding the final drug–drug interaction prediction results.

The framework of our model is depicted in Fig. 1. In the GNN encoder, we use RDKit to convert drug SMILES sequences into molecular graphs, which are then encoded using GIN. Subsequently, we employ two Transformer-like encoders: a self-attention mechanism encoder and an interaction attention mechanism encoder, both equipped with learnable pattern vectors, to compress the graphs into *M* representative vectors. Within the GSAT encoder, cosine similarity is computed for each pair of representative vectors from two drugs, resulting in an M×M similarity matrix. After flattening the similarity matrix, the resulting vectors encapsulate rich features of drug interactions. Simultaneously, in the interaction attention mechanism encoder GIAT, drug pair features are obtained after the interaction attention mechanism to derive feature matrices $O_{1}$ and $O_{2}$. These matrices undergo average pooling and standardization, yielding vector pairs containing interaction features of substructures between drug pairs. Finally, the vectors obtained from the GSAT and GIAT encoders are concatenated and input into an MLP （Multilayer Perceptron） layer to produce the final prediction.

### 2.4 GSAT encoder

**Figure 2.**
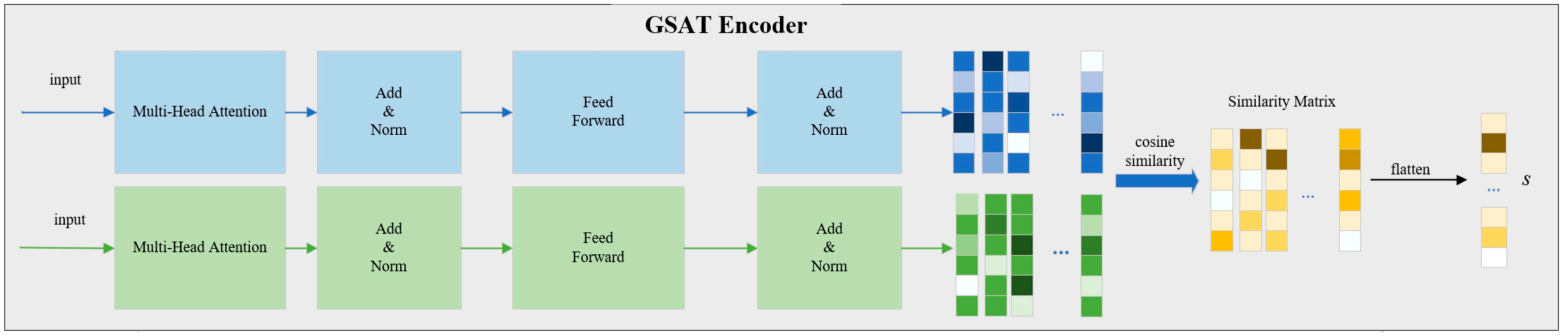
The detail of the GSAT Encoder. The drug features obtained through the GNN encoder are processed by the self-attention mechanism to generate different feature matrices between the drugs. The cosine similarity matrix *S* is then computed, and after flattening, it results in the vector *s* that contains the interaction features.

As illustrated in Fig. 2, the GSAT module utilizes a self-attention mechanism to derive self-attention scores between individual queries and keys, subsequently using these scores to distill information from the corresponding values. The formulation can be articulated as follows:

(1) $$\begin{array}{c} Q=Q_{0}W_{Q},\;K=K_{0}W_{K},\;V=V_{0}W_{V}, \end{array}$$

(2) $$\begin{array}{c} A=\text{softmax}\left(\frac{QK^{⊤}}{\sqrt{d}}\right), \end{array}$$

(3) $$\begin{array}{c} O=\text{ReLU}\left((Q+AV)W_{0}\right), \end{array}$$

where *Q*, *K*, and *V* denote the queries, keys, and values, respectively, with *d* representing the embedding dimension. The function ReLU(·) denotes the Rectified Linear Unit activation function. $W_{Q}$, $W_{K}$, $W_{V}$, and $W_{0}$ denote learnable weights. The *M* learnable queries (patterns) are initialized in $Q_{0}\in R^{M\times d}$ and are randomly initialized. We use the GNN-encoded node representations as our keys and values. $K_{0}$ and $V_{0}$ are represented by the following formulation:

(4) $$\begin{array}{c} K_{0}=V_{0}=[h_{1}^{\left(L\right)},h_{2}^{\left(L\right)},\ldots ,h_{N}^{\left(L\right)}], \end{array}$$

where *L* denotes the number of GNN layers, $h_{i}^{\left(L\right)}$ represents the representation of node *i* at the *L*-th layer, and *N* denotes the number of nodes. Ultimately, we obtain *M* representative vectors for each drug from Equation (3), corresponding to *M* substructure patterns.

(5) $$\begin{array}{c} {}[z_{1},z_{2},\ldots ,z_{M}]=O\in R^{M\times d}. \end{array}$$

Once we have obtained the representative vectors, cosine similarity is used to measure each pair of representative vectors from the two drugs, thereby generating a similarity matrix $S_{ij}\in R^{M\times M}$. The formulation of computing cosine similarity can be described as follows:

(6) $$\begin{array}{c} S_{ij}=\frac{{\left(Z_{i}^{1}\right)}^{⊤}Z_{j}^{2}}{||Z_{i}^{1}|{|}_{2}||Z_{j}^{2}|{|}_{2}}\in [-1,1]. \end{array}$$

The similarity module non-parametrically characterizes interactions among the substructures of the two drugs. The elements of $S_{ij}$ denote the strength of interaction, thereby enhancing the interpretability of prediction outcomes.

### 2.5 GIAT encoder

**Figure 3.**
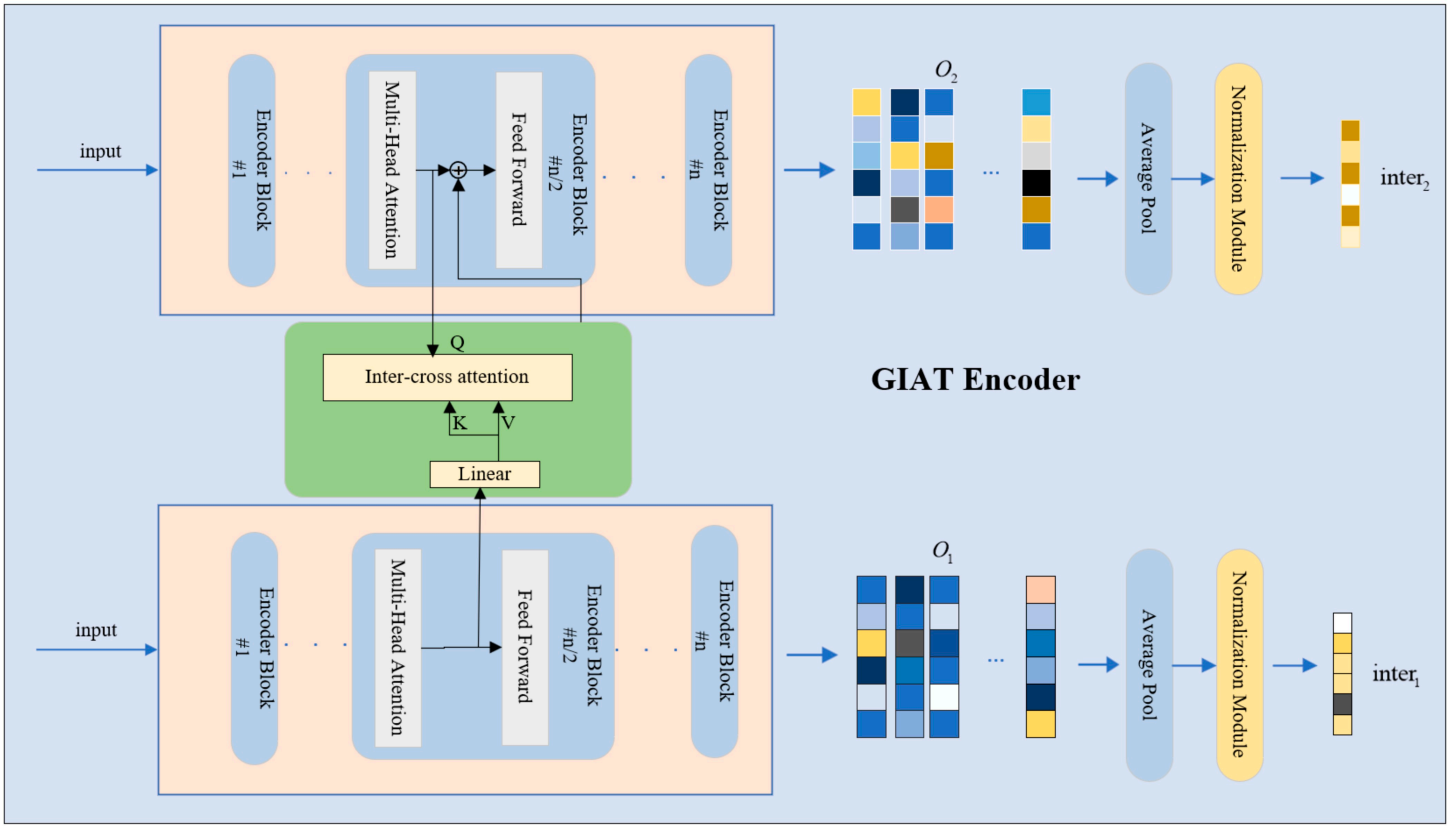
The detailed operational principle of the GIAT encoder employs a cross-attention mechanism to process drug matrices derived from the GNN encoder, thereby producing distinct feature matrices denoted as $O_{1}$ and $O_{2}$ for each drug pair. Subsequently, through average pooling and normalization, interaction vectors ${\text{inter}}_{1}$ and ${\text{inter}}_{2}$ are computed for these drug pairs.

To more closely examine the substructural effects in drug pairs, we use an encoder with a cross-attention mechanism to learn the interaction patterns between drug pairs, as shown in Fig. 3. The formulation can be described as follows:

The representations $x_{1}$ and $x_{2}$ are derived from the GIN-encoded representations:

(7) $$\begin{array}{c} x_{1}=\text{GIN}(G_{1}),x_{2}=\text{GIN}(G_{2}), \end{array}$$

where $G_{1}$ and $G_{2}$ denote the molecular graphs of the respective drugs. The GIN layer is formulated as:

(8) $$\begin{array}{c} h_{i}^{\left(l+1\right)}=h_{i}^{\left(l\right)}+\text{MLP}\left(\sum_{j\in N(i)}h_{j}^{\left(l\right)}\right), \end{array}$$

where N(i) denotes the neighbors of node *i*, and MLP represents a multi-layer perceptron.

We first calculate the keys and values of each drug: $K_{1}$ and $V_{1}$ are then converted into dense batch formats, incorporating batch indices for alignment. Similarly, $K_{2}$ and $V_{2}$ are processed into dense batches.

(9) $$\begin{array}{c} K_{1}=W_{1}^{K}(x_{1}),\;V_{1}=W_{1}^{V}(x_{1}), \end{array}$$

(10) $$\begin{array}{c} K_{2}=W_{2}^{K}(x_{2}),\;V_{2}=W_{2}^{V}(x_{2}). \end{array}$$

The query generation of each drug can be described as follows: queries for each drug are generated by tiling a set of learnable query patterns and transforming them through a weight matrix $W^{Q}$.

(11) $$\begin{array}{c} Q_{1}=W^{Q}(\text{tile}(Q_{\text{tile}}(K_{1}.\text{size}(0,1,1)))), \end{array}$$

(12) $$\begin{array}{c} Q_{2}=W^{Q}(\text{tile}(Q_{\text{tile}}(K_{2}.\text{size}(0,1,1)))), \end{array}$$

where $K_{1}$ represents the result of the linear transformation $W_{1}^{K}$ applied to input $x_{1}$, which are the keys for the first drug. $K_{1}.\text{size}(0,1,1)$ denotes the size of $K_{1}$ along its dimensions after incorporating the batch information batch1 to ensure it is dense. $Q_{\text{tile}}$ duplicates the query matrix *Q* along its first dimension to align with the batch size of $K_{1}$. $Q_{1}$ denotes the resulting query matrix after *Q* undergoes a linear transformation $W_{Q}$ following the tiling process. Similarly, for the second drug, $K_{2}$ represents the result of the linear transformation $W_{2}^{K}$ applied to input $x_{2}$, which are the keys for the second drug. $K_{2}.\text{size}(0,1,1)$ denotes the size of $K_{2}$ along its dimensions after incorporating the batch information batch2. $Q_{\text{tile}}$ duplicates the query matrix *Q* along its first dimension to align with the batch size of $K_{2}$. $Q_{2}$ denotes the resulting query matrix after *Q* undergoes a linear transformation $W_{Q}$ following the tiling process.

Attention scores are computed between the queries of one drug and the keys of the other drug, and vice versa. The formula for computing attention scores is as follows:

(13) $$\begin{array}{c} A_{1}=\text{softmax}\left(\frac{Q_{1}K_{2}^{⊤}}{\sqrt{d}}\right), \end{array}$$

where *d* is the dimensionality of the key vectors. Furthermore, a threshold parameter λ is applied to select the top attention values, filtering out less relevant interactions:

(14) $$\begin{array}{c} \mathrm{Mas}k_{1}=A_{1}⩾\lambda ,\mathrm{Mas}k_{2}=A_{2}⩾\lambda . \end{array}$$

The final outputs are calculated by multiplying the filtered attention matrices by the value matrices of the opposite drug and applying a linear transformation followed by a ReLU activation:

(15) $$\begin{array}{c} O_{1}=\text{ReLU}\left(W_{O}((Q_{1}+\mathrm{Mas}k_{1}V_{2}))\right), \end{array}$$

(16) $$\begin{array}{c} O_{2}=\text{ReLU}\left(W_{O}((Q_{2}+\mathrm{Mas}k_{2}V_{1}))\right). \end{array}$$

### 2.6 Normalization module

To mitigate overfitting after obtaining $O_{1}$ and $O_{2}$, we perform pooling and normalization on $O_{1}$ and $O_{2}$. The computations are as follows:

(17) $$\begin{array}{c} {\bar{O}}_{1}=\text{AvgPool}(O_{1}),\;{\bar{O}}_{2}=\text{AvgPool}(O_{2}), \end{array}$$

where AvgPool(·) denotes the average pooling activation function.

Then, we apply normalization to ${\bar{O}}_{1}$ and ${\bar{O}}_{2}$. The computations are as follows:

(18) $$\begin{array}{c} {\text{inter}}_{1}=\frac{{\bar{O}}_{1}-\mu_{{\bar{O}}_{1}}}{\sigma_{{\bar{O}}_{1}}},\;{\text{inter}}_{2}=\frac{{\bar{O}}_{2}-\mu_{{\bar{O}}_{2}}}{\sigma_{{\bar{O}}_{2}}}. \end{array}$$

The variables ${\text{inter}}_{1}$ and ${\text{inter}}_{2}$ represent the interaction vector representations of different drugs. The symbols $\mu_{{\bar{O}}_{1}}$ and $\mu_{{\bar{O}}_{2}}$ represent the mean values of ${\bar{O}}_{1}$ and ${\bar{O}}_{2}$, respectively. The symbols $\sigma_{{\bar{O}}_{1}}$ and $\sigma_{{\bar{O}}_{2}}$ represent the standard deviations of ${\bar{O}}_{1}$ and ${\bar{O}}_{2}$, respectively.

### 2.7 MLP layer

Finally, the similarity matrix *S* is flattened, concatenated with the representations of the two drugs, and fed into an MLP prediction layer.

(19) $$\begin{array}{c} s=\text{flatten}(S), \end{array}$$

(20) $$\begin{array}{c} y=\text{MLP}({\text{inter}}_{1}\;∥\;{\text{inter}}_{2}\;∥\;s), \end{array}$$

where ∥ denotes concatenation, and ${\text{inter}}_{1}$ and ${\text{inter}}_{2}$ denote the representations of drug pairs obtained from the GIAT encoder. We utilize Binary Cross Entropy loss as our loss function, formulated as follows:

(21) $$\begin{array}{c} L=-\frac{1}{n}\sum_{i=1}^{n}\left(y_{i}^{\prime }\;\text{log}\;\sigma (y_{i})+(1-y_{i}^{\prime })\;\text{log}(1-\sigma (y_{i}))\right), \end{array}$$

where $y_{i}$ is the output of the *i*-th drug pair, $y_{i}^{\prime }\in \{0,1\}$ is the label of the *i*-th drug pair, σ(·) is the sigmoid function, and *n* is the number of drug pairs.

## 3 Experimental result

### 3.1 Evaluation metrics and experimental setup

In our experimental evaluation, we have chosen four metrics area under the receiver operating characteristic curve (AUROC), average precision (AP), F1-score (F1), and accuracy (ACC) to comprehensively assess the model’s performance in predicting DDIs. To ensure the reliability of our results and mitigate the impact of random variability, each experiment is conducted five times, and we report the mean values of these metrics. For a detailed description of these four metrics, please refer to Supplementary Section S3.

We conducted all experiments on an Ubuntu release 20.04 system utilizing the NVIDIA A40-PCIE GPU card with 48 GB of memory. To ensure equitable performance comparisons, all models were implemented in PyTorch. Our model was trained for 300 epochs, with a learning rate of 0.001 for the first 150 epochs and 0.0001 for the subsequent 150 epochs. The batch size was set to 512, and the node embedding dimension was fixed at 128. We utilized 60 representative vectors *M* and employed five layers in the GIN structure. Additionally, we initialized the DDI using Xavier initialization (Glorot and Bengio 2010) and optimized the model parameters using the Adam optimizer (Kingma and Ba 2014).

### 3.2 Comparison model description

In comparative experiments, we compare ten state-of-the-art methods: MR-GNN, GCN-BMP, EPGCN-DS, DeepDrug, MIRACLE, SSI-DDI, CSGNN, DeepDDS, DSN-DDI, and HTCL. Here are some brief introductions:

- MR-GNN (Xu *et al.* 2019) employs a multi-resolution architecture to capture local features of each graph and extract interaction features between pairwise graphs.
- GCN-BMP (Chen *et al.* 2020) leverages GNN for DDI prediction by employing an end-to-end graph representation learning framework.
- EPGCN-DS (Sun *et al.* 2020) detects DDIs from molecular structures using an encoder with expressive GCN layers and a decoder that outputs the probability of DDI.
- DeepDrug (Cao *et al.* 2020) employs residual graph convolutional networks (RGCNs) along with convolutional networks (CNNs) to enhance the accuracy of DDI prediction.
- MIRACLE (Wang *et al.* 2021) offers a multi-view framework that simultaneously captures the intra-view molecular structure and the inter-view DDIs between molecules.
- SSI-DDI (Nyamabo *et al.* 2021) deconstructs the DDI prediction task between two drugs to pinpoint pairwise interactions among their respective substructures.
- CSGNN (Zhao *et al.* 2021) incorporates a mix-hop neighborhood aggregator into a GNN to capture high-order dependencies in DDI networks and utilizes a contrastive self-supervised learning task as a regularizer.
- DeepDDS (Wang *et al.* 2022) is a deep learning model that employs GNNs and an attention mechanism to identify synergistic drug combinations.
- DSN-DDI (Li *et al.* 2023) is a dual-view drug representation learning network designed to learn drug substructures from individual drugs and drug pairs simultaneously.
- HTCL-DDI (Zhang *et al.* 2023) is a hierarchical triple-view contrastive learning framework for predicting DDIs.

### 3.3 Model performance comparison

In our study, we compared MSDAFL with 10 competitive DDI prediction models across three datasets of varying scales, using three widely adopted evaluation metrics (AUROC, AUPRC, and F1) to assess their predictive performance. Table 1 summarizes the experimental outcomes of MSDAFL and other baseline methods across these datasets on training, validation and testing sets in a ratio of 6:2:2. MSDAFL consistently demonstrated superior performance across multiple evaluation metrics and datasets. On the ZhangDDI dataset, HTCL-DDI achieved superior results in AP and F1 metrics by leveraging diverse view relationships and integrating multi-view features for DDI prediction. On the ChCh-Miner and DeepDDI datasets, MSDAL showed substantial improvements compared to HTCL. For instance, on DeepDDI, MSDAFL improved AUROC by approximately 5% and ACC by around 6%. SSI-DDI deconstructs the DDI prediction task between drug pairs to identify pairwise interactions among their respective substructures. In contrast, DSN-DDI is a dual-view drug representation learning network designed to simultaneously learn drug substructures from individual drugs and drug pairs. MSDAFL comprehensively outperforms SSI-DDI and DSN-DDI across all metrics on the three datasets. As a multi-attention mechanism network framework, MSDAFL effectively applies various attention mechanisms to predict DDIs, processing drugs from different interaction perspectives to achieve robust and diverse drug representations. The outstanding performance of MSDAFL across these datasets underscores the potential of interactive attention mechanisms in capturing critical features relevant to DDIs.

**Table 1.** Comparison of MSDAFL with other DDI prediction methods on training, validation, and testing sets in a ratio of 6:2:2.^a^

Dataset	Metric	MR-GNN	GCN-BMP	EPGCN-DS	DeepDrug	MIRACLE	SSI-DDI	CSGNN	DeepDDS	DSN-DDI	HTCL-DDI	MSDAFL
**ZhangDDI**	AUROC	0.9618 ± 0.0025	0.8442 ± 0.0121	0.9083 ± 0.0066	0.9535 ± 0.0020	0.9644 ± 0.0035	0.9314 ± 0.0029	0.9171 ± 0.0009	0.9320 ± 0.0023	0.9113 ± 0.0015	0.9858 ± 0.0021	**0.9874 ± 0.0011**
	AP	0.9263 ± 0.0030	0.8020 ± 0.0157	0.8896 ± 0.0088	0.9233 ± 0.0023	0.9309 ± 0.0053	0.9209 ± 0.0039	0.8902 ± 0.0073	0.9208 ± 0.0031	0.8642 ± 0.0030	0.9706 ± 0.0038	**0.9707 ± 0.0018**
	F1	0.8293 ± 0.0081	0.7186 ± 0.0271	0.8007 ± 0.0086	0.8289 ± 0.0027	0.8516 ± 0.0027	0.8196 ± 0.0124	0.8360 ± 0.0073	0.8279 ± 0.0042	0.8768 ± 0.0040	**0.9219 ± 0.0056**	0.9005 ± 0.0014
	ACC	0.9190 ± 0.0050	0.7578 ± 0.0107	0.8240 ± 0.0104	0.8567 ± 0.0033	0.9316 ± 0.0016	0.8535 ± 0.0050	0.8414 ± 0.0045	0.8563 ± 0.0028	0.8665 ± 0.0046	**0.9659 ± 0.0024**	0.9533 ± 0.0026
**ChCh-Mainer**	AUROC	0.9311 ± 0.0036	0.7865 ± 0.0056	0.9423 ± 0.0071	0.9838 ± 0.0010	0.9620 ± 0.0079	0.9809 ± 0.0014	0.9768 ± 0.0010	0.9710 ± 0.0018	0.9669 ± 0.0020	0.9906 ± 0.0015	**0.9934 ± 0.0031**
	AP	0.9595 ± 0.0019	0.8631 ± 0.0054	0.9680 ± 0.0040	0.9916 ± 0.0005	0.9950 ± 0.0011	0.9897 ± 0.0006	0.9756 ± 0.0016	0.9851 ± 0.0008	0.9634 ± 0.0027	0.9987 ± 0.0002	**0.9991 ± 0.0007**
	F1	0.8813 ± 0.0072	0.8087 ± 0.0092	0.8941 ± 0.0066	0.9467 ± 0.0026	0.9455 ± 0.0066	0.9398 ± 0.0034	0.9247 ± 0.0022	0.9221 ± 0.0063	0.8812 ± 0.0064	0.9748 ± 0.0019	**0.9932 ± 0.0044**
	ACC	0.8503 ± 0.0062	0.7307 ± 0.0080	0.8664 ± 0.0098	0.9318 ± 0.0035	0.9077 ± 0.0011	0.9219 ± 0.0048	0.9254 ± 0.0017	0.9038 ± 0.0064	0.8889 ± 0.0042	0.9561 ± 0.0032	**0.9830 ± 0.0012**
**DeepDDI**	AUROC	0.9335 ± 0.0017	0.7719 ± 0.0063	0.8593 ± 0.0024	0.9174 ± 0.0014	0.9276 ± 0.0038	0.9179 ± 0.0048	0.9401 ± 0.0025	0.9438 ± 0.0063	0.9322 ± 0.0010	0.9449 ± 0.0020	**0.9974 ± 0.0011**
	AP	0.9456 ± 0.0009	0.8170 ± 0.0060	0.8872 ± 0.0012	0.9299 ± 0.0018	0.9677 ± 0.0018	0.9347 ± 0.0044	0.9417 ± 0.0030	0.9568 ± 0.0056	0.9287 ± 0.0015	0.9741 ± 0.0010	**0.9987 ± 0.0012**
	F1	0.9007 ± 0.0049	0.8010 ± 0.0026	0.8486 ± 0.0038	0.8939 ± 0.0009	0.9354 ± 0.0070	0.8823 ± 0.0049	0.8601 ± 0.0063	0.9127 ± 0.0054	0.8560 ± 0.0015	0.9478 ± 0.0027	**0.9911 ± 0.0043**
	ACC	0.8754 ± 0.0043	0.7294 ± 0.0049	0.8022 ± 0.0039	0.8628 ± 0.0012	0.9033 ± 0.0098	0.8538 ± 0.0059	0.8633 ± 0.0036	0.8887 ± 0.0068	0.8541 ± 0.0009	0.9208 ± 0.0037	**0.9866 ± 0.0024**

a The superior results are emphasized in bold, while the second-best results are underlined.

In addition, we also compare MSDAFL and other baseline methods across these datasets on training, validation and testing sets in a ratio of 8:1:1. The results are shown in the Table 2. The MSADFL model demonstrates improved performance on the ZhangDDI dataset. For the ChCh-Miner and DeepDDI datasets, the model’s performance remains stable across all four metrics. This stability highlights the strong capabilities of our model’s self-attention mechanism and interaction attention. In contrast, the HTCL-DDI model shows a decline in performance across all datasets, particularly on the DeepDDI dataset. This decline underscores the limitations of the approach that leverages existing drug interaction networks, where drugs are nodes and their interactions are edges, which can negatively impact drug interaction prediction. Our model outperforms others across all three datasets, indicating that it surpasses other models in predictive performance. Furthermore, to assess the generalization performance of the MSADFL model, we further conduct cross-datum study experiments on three datasets: ZhangDDI, DeepDDI, and ChCh-Miner. The experimental results of MSADFL and HTCL-DDI are presented in Supplementary Tables S2 and S3, respectively. Compared to previous experiments across three datasets with varying scales, the prediction performances of MSDAFL and HTCL-DDI are declined. Notably, when DeepDDI is used as the training set and ChCh-Miner as the test set, the AUC reaches 0.8227, highlighting the model’s robust generalization ability.

**Table 2.** Comparison of MSDAFL with other DDI prediction methods on training, validation and testing sets in a ratio of 8:1:1.^a^

Dataset	Metric	MR-GNN	GCN-BMP	EPGCN-DS	DeepDrug	MIRACLE	SSI-DDI	CSGNN	DeepDDS	DSN-DDI	HTCL-DDI	MSDAFL
**ZhangDDI**	AUROC	0.9434 ± 0.0015	0.8512 ± 0.0159	0.9043 ± 0.0018	0.9477 ± 0.0009	0.8914 ± 0.0021	0.9279 ± 0.0025	0.9871 ± 0.0012	0.9212 ± 0.0034	0.7113 ± 0.0035	0.9882 ± 0.0031	**0.9912 ± 0.0012**
	AP	0.9133 ± 0.0052	0.8220 ± 0.0201	0.8996 ± 0.0065	0.9351 ± 0.0042	0.9312 ± 0.0043	0.8913 ± 0.0074	0.9712 ± 0.0087	0.9031 ± 0.0012	0.6756 ± 0.0045	0.9514 ± 0.0048	**0.9907 ± 0.0033**
	F1	0.8463 ± 0.0074	0.7086 ± 0.0154	0.7958 ± 0.0046	0.8473 ± 0.0012	0.9282 ± 0.0017	0.8412 ± 0.0034	0.8731 ± 0.0034	0.8412 ± 0.0075	0.6712 ± 0.0031	0.9219 ± 0.0056	**0.9405 ± 0.0014**
	ACC	0.8884 ± 0.0099	0.7675 ± 0.0098	0.8212 ± 0.0064	0.8753 ± 0.0055	0.9391 ± 0.0014	0.8132 ± 0.0062	0.8992 ± 0.0014	0.8812 ± 0.0034	0.6513 ± 0.0034	**0.9568 ± 0.0031**	0.9553 ± 0.0012
**ChCh-Mainer**	AUROC	0.9451 ± 0.0009	0.7762 ± 0.0081	0.9015 ± 0.0064	0.9902 ± 0.0020	0.9540 ± 0.0012	0.9809 ± 0.0014	0.9912 ± 0.0035	0.9717 ± 0.0073	0.9218 ± 0.0032	0.9836 ± 0.0021	**0.9964 ± 0.0021**
	AP	0.9605 ± 0.0069	0.8351 ± 0.0071	0.9590 ± 0.0013	0.9874 ± 0.0015	0.9810 ± 0.0023	0.9897 ± 0.0006	0.9831 ± 0.0034	0.9881 ± 0.0012	0.9112 ± 0.0032	0.9931 ± 0.0012	**0.9988 ± 0.0009**
	F1	0.9023 ± 0.0122	0.7187 ± 0.0054	0.8741 ± 0.0046	0.9323 ± 0.0051	0.9712 ± 0.0062	0.9398 ± 0.0034	0.9271 ± 0.0022	0.9331 ± 0.0051	0.8432 ± 0.0014	0.9701 ± 0.0021	**0.9911 ± 0.0021**
	ACC	0.8653 ± 0.0058	0.7543 ± 0.0019	0.8061 ± 0.0074	0.9216 ± 0.0071	0.9534 ± 0.0032	0.9219 ± 0.0048	0.8912 ± 0.0064	0.9151 ± 0.0043	0.8465 ± 0.0012	0.9513 ± 0.0013	**0.9850 ± 0.0022**
**DeepDDI**	AUROC	0.9402 ± 0.0041	0.7412 ± 0.0085	0.8393 ± 0.0054	0.9062 ± 0.0043	0.8965 ± 0.0023	0.9429 ± 0.0025	0.9531 ± 0.0035	0.9056 ± 0.0019	0.7412 ± 0.0031	0.9152 ± 0.0022	**0.9954 ± 0.0014**
	AP	0.9514 ± 0.0065	0.8023 ± 0.0054	0.8566 ± 0.0066	0.9444 ± 0.0045	0.9471 ± 0.0044	0.9213 ± 0.0074	0.9411 ± 0.0030	0.9217 ± 0.0085	0.7213 ± 0.0041	0.8921 ± 0.0014	**0.9937 ± 0.0022**
	F1	0.9052 ± 0.0053	0.7745 ± 0.0056	0.8214 ± 0.0061	0.8639 ± 0.0036	0.9352 ± 0.0065	0.8712 ± 0.0034	0.8355 ± 0.0019	0.8951 ± 0.0064	0.6060 ± 0.0015	0.8828 ± 0.0008	**0.9821 ± 0.0023**
	ACC	0.8873 ± 0.0074	0.6444 ± 0.0056	0.7023 ± 0.0059	0.8021 ± 0.0058	0.9042 ± 0.0086	0.8632 ± 0.0062	0.8413 ± 0.0023	0.8552 ± 0.0012	0.6634 ± 0.0017	0.8694 ± 0.0043	**0.9812 ± 0.0024**

a The superior results are emphasized in bold, while the second-best results are underlined.

### 3.4 Ablation experiment

The outstanding performance of MSDAFL stems from three carefully designed strategies: the cross-attention mechanism strategy between drug pairs, normalization of the interaction matrix, and self-attention mechanism strategy with cosine similarity. To ascertain the efficacy of each drug feature type, we conducted ablation experiments on the ZhangDDI dataset across these three configurations on training, validation and testing sets in a ratio of 6:2:2.The results are shown in the Supplementary Fig. S1, demonstrating the effectiveness of the module we proposed.

### 3.5 Parameter sensitivity

To investigate the influence of crucial parameters on prediction performance, we systematically vary these parameters and assessed their impact on the MSDAFL model’s efficacy using the ZhangDDI dataset on training, validation and testing sets in a ratio of 6:2:2. We analyze the batch size for model training, the parameter λ, and the number of GIN layers. By holding other parameters constant, we explore how varying key parameter settings impacted the performance of MSDAFL. As illustrated in the Supplementary Fig. S2, we investigated how these parameters affect model performance. Specifically, we found that the model performs optimally when the batch size is set to 512, λ to 0.75, and the number of GIN layers to 5.

### 3.6 Case study

To assess the practical utility of MSDAFL in real-world scenarios, we performed an analysis of clinical studies evaluating the prediction outcomes for four drug pairs using MSDAFL on the ZhangDDI testing set, as depicted in Supplementary Fig. S3. For the analysis of these four drugs, we can confirm the powerful performance of the MSDAFL model in predicting DDIs.

## 4 Discussion and conclusion

In this work, we introduce a molecular substructure-based dual attention feature learning framework for predicting DDIs. This framework integrates multiple attention mechanisms, including a self-attention encoder that extracts substructures from individual drugs and computes a cosine similarity matrix between the feature matrices of drug pairs. In the interactive attention encoding segment, we employ an interactive attention mechanism to investigate the strength of interactions between substructures of drug pairs, culminating in the regularization of the interactive feature matrix. Extensive experiments are conducted across three public datasets to evaluate the efficacy of our MSDAFL model and assess the contributions of its various modules. The findings decisively establish MSDAFL as a robust and promising tool for predicting DDIs, significantly contributing to medication safety and drug side effect research. Our study can be further advanced in three key domains: (i) by integrating heterogeneous biomedical information to augment representation learning, (ii) by expanding MSDAFL to more complex and practical application scenarios, and (iii) by supplementing with wet-lab experiments to further validate certain DDI prediction outcomes.

## Supplementary Material

btae596_Supplementary_Data

## Data Availability

Our code and data are available at: https://github.com/27167199/MSDAFL.
