# Supplementary material for: MSDAFL: molecular substructure-based dual attention feature learning framework for predicting drug–drug interactions
Source: Bioinformatics. 2024 Oct 9;40(10):btae596. doi: 10.1093/bioinformatics/btae596 (PMC11486503; doi:10.1093/bioinformatics/btae596)
Supplement: btae596_Supplementary_Data [file btae596_supplementary_data.pdf]

1 Dataset

According to Table S1, these three datasets represent small, medium, and large datasets based on drug quantity, effectively testing the generalization performance of MSDAFL.

Table S1: Summary of DDI datasets

Dataset	Number of Drugs	Number of DDIs
ZhangDDI	544	45,720
ChCh-Miner	997	21,486
DeepDDI	1,704	191,870

2 Node Feature Matrix

The following is a detailed process for obtaining the node feature matrix X used by our model.

2.1 Extracting Atomic Features

First, multiple key chemical properties are extracted from the structure of each molecule at the atomic level. These features include:

- **Atomic Symbol**: For example, element types such as Carbon (C) and Oxygen (O).
- **Number of Bonds (Degree)**: The number of chemical bonds an atom forms with other atoms.
- **Implicit Valence**: The number of implicit valence electrons of the atom.
- **Formal Charge**: The charge state of the atom.
- **Radical Electrons**: The number of radical electrons in the atom.
- **Hybridization State**: The hybrid orbital state of the atom (e.g., sp, sp2).
- **Total Number of Hydrogens**: The number of hydrogen atoms directly bonded to the atom.
- **Aromaticity**: Whether the atom is part of an aromatic ring system.

These features provide a unique chemical description for each atom, which will be used to construct feature vectors.

2.2 Building Feature Dictionaries

After extracting atomic features, a feature dictionary is built for each feature. The feature dictionary maps all possible values of the feature to a unique index. This step ensures that each atomic feature is standardized and can be easily encoded and compared in subsequent processing.

2.3 Feature Encoding

Once the feature dictionaries are constructed, the next step is to encode the feature values for each atom. Each atomic feature value is mapped to its corresponding index, and these indices are then converted into one-hot encoded vectors (i.e., high-dimensional vectors where only one position is set to 1, and the others are set to 0). This encoding method helps the model distinguish between different features while preserving the information of each feature.

2.4 Generating the Node Feature Matrix

For each atom, all the one-hot encoded vectors are concatenated to form a complete feature vector. These feature vectors then constitute the node feature matrix X of the molecular graph. Each row of the matrix X represents the features of an atom, covering all the chemical properties of the atom.

2.5 Saving and Utilizing the Node Feature Matrix

Finally, the node feature matrix X is saved along with the molecular edge information (i.e., the chemical bonds between atoms) for use in subsequent model training and prediction tasks. These features provide the necessary input data for models such as graph neural networks, supporting further analysis and prediction of molecular properties.This process ensures that the chemical properties of each atom are systematically encoded and represented in a manner that can be effectively analyzed and processed by machine learning models, enabling the molecular structure to be analyzed and utilized efficiently.

3 Comparison Metrics

These metrics are pivotal in evaluating the predictive accuracy and effectiveness of the model. The formulas for these metrics are specified as follows:

$$ACC = \frac{TP + TN}{TP + TN + FP + FN}, \tag{1}$$

$$F1 = 2 \times \frac{\text{Precision} \times \text{Recall}}{\text{Precision} + \text{Recall}}, \tag{2}$$

where TP and TN denote the number of true positives and true negatives correctly predicted by the model, while FP and FN denote the number of

false positives and false negatives, respectively. Additionally, AUROC and AP provide insights into the model's discriminatory ability across various decision thresholds and the precision-recall trade-off, respectively.

4 Ablation experiment

We perform three experiments without the cross-attention mechanism strategy between drug pairs, without normalizing the interaction matrix, and without the self-attention mechanism strategy with cosine similarity. As shown in Supplementary Figure S1, the model with combined features outperforms individual features. The performance of the model without normalization also decreases. When all three features are fused together, the model achieves optimal performance across all metrics, with an accuracy (ACC) score of 0.9533, AUROC score of 0.9874, AP score of 0.9707, and F1 score of 0.9005. The experiments demonstrate that each design plays a role in the final DDI prediction to some extent. The model incorporating all three features achieves superior predictive performance, underscoring its capability to comprehensively capture drug interaction characteristics. These findings highlight that the dual attention mechanism enhances drug representation and improves DDI prediction performance. From Figure S1, it can be observed that the performance of our model declines to varying degrees when any of these components is omitted. This demonstrates the effectiveness of our proposed innovations in predicting drug interactions.

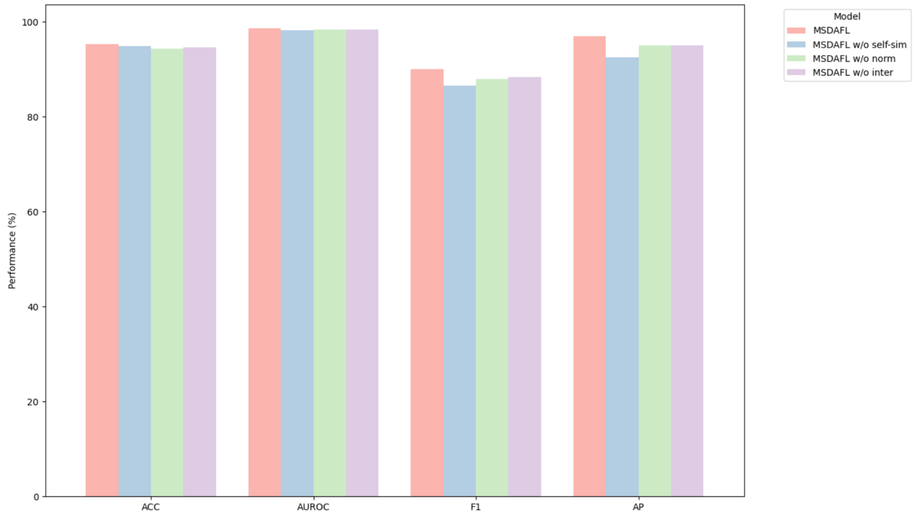

Figure S1: The comparative analysis of MSDAFL performance with various feature combinations on the ZhangDDI dataset. "Self-sim" denotes the self-attention mechanism using cosine similarity. "Norm" denotes the normalization of the inter-attention feature matrix. "Inter" denotes the inter-attention mechanism and normalization.

5 Parameter sensitivity

To identify the optimal batch size, we vary it across a range from 64 to 1024. As shown in Figure S2, when the batch size is set to 512, all three metrics reach their optimal values, and the model exhibits the best performance. Specifically, as the batch size increases, MSDAFL is able to extract more useful information. However, too large a batch size can increase noise and lead to a decline in performance, particularly when the batch size is 1024, where a significant drop in model performance is observed.

To explore the impact of the parameter λ on the experimental results, we set its value to 0.55, 0.65, 0.75, 0.85, 0.9, and 1, while keeping other parameters fixed. The performance of MSDAFL with different values of λ is shown in Figure S2(b). We find that the model performs best when the parameter is set to 0.75, while at very low or very high values of λ, the model performance significantly deteriorates. This could be because, when λ is set too low, it filters out substructures that affect drug interactions, whereas when λ is set to 1, the model retains substructures that have a minor role in drug interactions.

We keep all other parameters constant to investigate how the number of GIN layers affects the model performance. The model performs best when the number of GIN layers is set to 5, as shown in Figure S2(c). Each additional GIN layer enables the network to capture a broader range of neighborhood information, theoretically allowing for a better understanding of the context and relationships of nodes within the graph. However, when the number of GIN layers is set to 6, there is a substantial decline in performance, as the features of the nodes might overly blend with their neighbors, causing a decrease in the distinctiveness of features among different nodes.

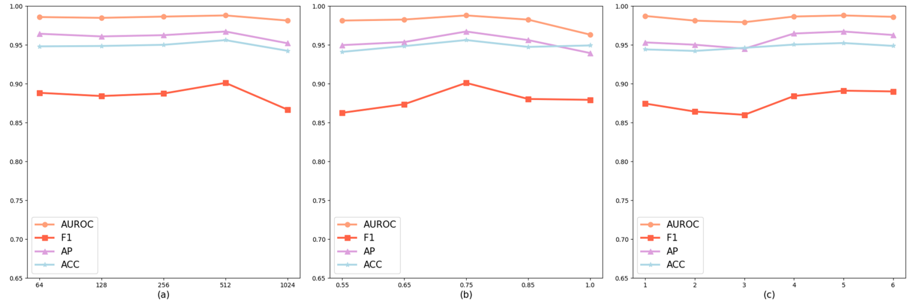

Figure S2: Performance comparison of the proposed MSDAFL with different model training batch sizes, λ, and GIN layers. The figure (a) shows the impact of different batch sizes on model prediction performance. The figure (b) shows the impact of different λ parameter on model prediction performance. The figure (c) shows the impact of different GIN layers on model prediction performance.

6 Cross-datum study

To assess the generalization performance of the MSADFL model, we conduct experiments on three datasets: ZhangDDI, DeepDDI and ChCh-Miner. In addition, we compare our method with second best method (HTCL-DDI). In each experiment, one dataset serves as the training and validation sets, while the remaining two are used as test sets. The experimental results of MSADFL and HTCL-DDI are presented in the Tables S2 and S3, respectively.

**Train-Test Dataset**	**ACC**	**AUC**	**F1**	**AP**
Deep-Miner	0.7824±0.0154	0.8227±0.0076	0.8328±0.0034	0.8604±0.0085
Deep-Zhang	0.6071±0.0087	0.5992±0.0042	0.4151±0.0121	0.4916±0.0064
Miner-Zhang	0.6209±0.0034	0.6464±0.0030	0.5987±0.0073	0.5034±0.0124
Miner-Deep	0.6456±0.0039	0.5913±0.0062	0.7557±0.0036	0.6467±0.0012
Zhang-Miner	0.6802±0.0084	0.7518±0.0137	0.7396±0.0094	0.8547±0.0059
Zhang-Deep	0.6048±0.0073	0.5812±0.0071	0.7302±0.0064	0.6733±0.0041
Average value	0.6568±0.0078	0.6654±0.0069	0.6787±0.0070	0.6717±0.0064

Table S2: Performance metrics of MSDAFL across different train-test dataset combinations. "Zhang" represents the ZhangDDI dataset, "Deep" represents the DeepDDI dataset, "Miner" represents the ChCh-Miner dataset, "Average value" represents the average value of all train-test dataset combinations.

**Train-Test Dataset**	**ACC**	**AUC**	**F1**	**AP**
Deep-Miner	0.7232±0.0103	0.7586±0.0076	0.7845±0.0131	0.7919±0.0079
Deep-Zhang	0.5526±0.0212	0.5888±0.0098	0.5499±0.0056	0.4640±0.0117
Miner-Zhang	0.5821±0.0081	0.6160±0.0117	0.5297±0.0032	0.5032±0.0108
Miner-Deep	0.7205±0.0141	0.7134±0.0042	0.7816±0.0029	0.7173±0.0063
Zhang-Miner	0.7266±0.0133	0.7498±0.0086	0.5296±0.0173	0.6030±0.0058
Zhang-Deep	0.5749±0.0044	0.5865±0.0106	0.7023±0.0031	0.8184±0.0111
Average value	0.6466±0.0119	0.6688±0.0087	0.6402±0.0075	0.6496±0.0089

Table S3: Performance metrics of HTCL-DDI across different train-test dataset combinations. "Zhang" represents the ZhangDDI dataset, "Deep" represents the DeepDDI dataset, "Miner" represents the ChCh-Miner dataset, "Average value" represents the average value of all train-test dataset combinations.

Compared to previous experiments across three datasets with varying scales, the prediction performances of MSDAFL and HTCL-DDI are declined. This decrease is primarily due to three datasets are constructed different research groups, and the varying sizes and data distributions of the three datasets, which contribute to these declinations. However, as shown in the Tables S2 and S3, the MSADFL model exhibits strong predictive capabilities on medium to large-sized datasets. Notably, when DeepDDI is used as the training set and ChCh-Miner as the test set, the AUC reaches 0.8227, highlighting the model's robust generalization ability. In addition, the average values of four metrics on all train-test dataset combinations also show that MSDAFL is superior with HTCL-DDI.

7 Case study

Our model has conducted research on the four predicted drug pairs, with the specific analysis as follows:

(1) As depicted in Fig. S3(A), the probability of a pharmacokinetic or pharmacodynamic interaction between Phenelzine and Miglustat appears to be negligible. Phenelzine is a monoamine oxidase inhibitor (MAOI) primarily used to treat depression. It functions by inhibiting the activity of monoamine oxidase, an enzyme responsible for breaking down neurotransmitters such as serotonin, norepinephrine, and dopamine, thereby increasing their levels and alleviating depressive symptoms [12]. Miglustat, on the other hand, is a medication used to treat Gaucher disease and Niemann-Pick disease type C. It functions by inhibiting glucosylceramide synthase, thereby reducing the synthesis of glycosphingolipids [7]. Unlike Phenelzine, Miglustat has a relatively low potential for drug interactions since it primarily acts through enzyme inhibition and undergoes minimal metabolism in the human body.

(2) Based on the depiction in Fig. S3(B), Caffeine and Isosorbide Dinitrate are unlikely to demonstrate significant interaction.Caffeine acts as a central nervous system stimulant by antagonizing adenosine receptors, which promotes the release of neurotransmitters such as dopamine and norepinephrine, enhancing cortical excitability [8, 9]. Conversely, Isosorbide Dinitrate is a nitrate drug utilized as a vasodilator to alleviate symptoms of angina. It achieves this by relaxing vascular smooth muscle through the release of nitric oxide, leading to dilation of both veins and arteries, which reduces cardiac workload and coronary artery resistance [11]. Given that Caffeine primarily influences the central nervous system and kidneys, while Isosorbide Dinitrate acts specifically on vascular smooth muscle through the cardiovascular system, their pharmacological pathways do not intersect or compete [3, 6]. Furthermore, their distinct metabolic and excretion pathways minimize the potential for drug interactions.

(3) As illustrated in Fig. S3(C),Acenocoumarol and Oxaprozin are susceptible to interacting with each other.Acenocoumarol, Acenocoumarol, an oral anticoagulant belonging to the coumarin family, inhibits vitamin K epoxide reductase, leading to a decrease in the production of vitamin K-dependent clotting factors, such as prothrombin [14]. This effect extends clotting time, making it useful in preventing and managing blood clots in conditions like deep vein thrombosis and pulmonary embolism [15]. On the other hand, Oxaprozin, a nonsteroidal anti-inflammatory drug (NSAID), primarily alleviates pain and inflammation associated with osteoarthritis and rheumatoid arthritis by inhibiting cyclooxygenase (COX) enzymes, particularly COX-1 and COX-2, thereby reducing prostaglandin production, which is involved in inflammation and pain [4]. When used concurrently, Acenocoumarol and Oxaprozin pose an increased risk of bleeding due to their cumulative effects on blood clotting mechanisms [1]. NSAIDs like Oxaprozin may interfere with the antiplatelet properties of oral anticoagulants, potentially prolonging bleeding time and heightening bleeding risk.

(4) Dronabinol, a synthetic cannabinoid, primarily targets brain cannabi-

noid receptors (CB1 receptors) [10]. It is prescribed to alleviate chemotherapy-induced nausea, vomiting, and appetite loss, particularly prevalent in patients undergoing chemotherapy or afflicted with AIDS [2]. Methoxamine, an α_1-adrenergic receptor agonist used as a vasoconstrictor, stimulates α_1 receptors to contract vascular smooth muscle, primarily indicated for treating hypotension or shock [5].As indicated by Fig. S3(D), Dronabinol and Methoxamine frequently interact with each other. Concurrent administration of Dronabinol and Methoxamine may enhance the vasoconstrictive effects of Methoxamine, potentially leading to elevated blood pressure and other adverse cardiovascular effects [13].

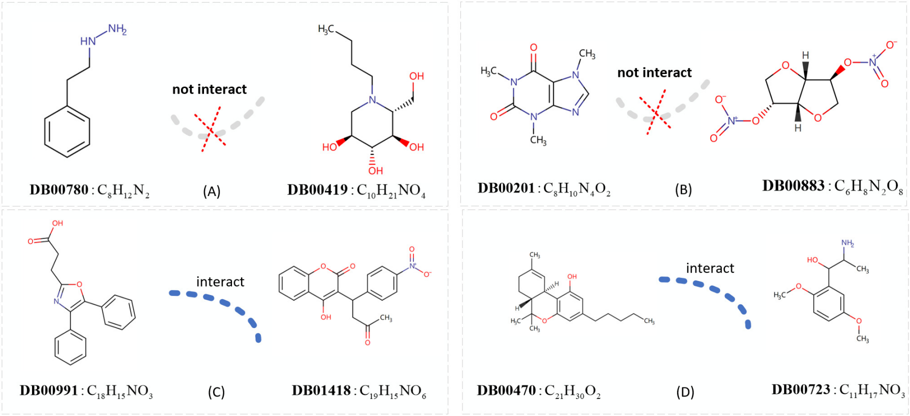

Figure S3: Case study of DDI prediction results: (A)The likelihood of interaction between Phenelzine and Miglustat is minimal. (B)Caffeine and Isosorbide Dinitrate are unlikely to exhibit significant interaction. (C)Acenocoumarol and Oxaprozin are prone to interacting with each other. (D)The interaction between Dronabinol and Methoxamine is frequently observed.

References

[1] Walter Ageno, Alexander S Gallus, Ann Wittkowsky, Mark Crowther, Elaine M Hylek, and Gualtiero Palareti. Oral anticoagulant therapy: antithrombotic therapy and prevention of thrombosis: American college of chest physicians evidence-based clinical practice guidelines. *Chest*, 141(2):e44S–e88S, 2012.

[2] Melissa E Badowski and Paa Kwesi Yanful. Dronabinol oral solution in the management of anorexia and weight loss in aids and cancer. *Therapeutics and clinical risk management*, pages 643–651, 2018.

[3] Robert T Cole, Andreas P Kalogeropoulos, Vasiliki V Georgiopoulou, Mihai Gheorghiade, Arshed Quyyumi, Clyde Yancy, and Javed Butler. Hydralazine and isosorbide dinitrate in heart failure: historical perspective, mechanisms, and future directions. *Circulation*, 123(21): 2414–2422, 2011.

[4] Neal M Davies. Clinical pharmacokinetics of oxaprozin. *Clinical pharmacokinetics*, 35(6): 425–436, 1998.

[5] Vanessa Erdmann, Torsten Sehl, Ilona Frindi-Wosch, Robert C Simon, Wolfgang Kroutil, and Dorte Rother. Methoxamine synthesis in a biocatalytic 1-pot 2-step cascade approach. *Acs Catalysis*, 9(8):7380–7388, 2019.

[6] Diederick E Grobbee, Eric B Rimm, Edward Giovannucci, Graham Colditz, Meir Stampfer, and Walter Willett. Coffee, caffeine, and cardiovascular disease in men. *New England Journal of Medicine*, 323(15):1026–1032, 1990.

[7] Paul L McCormack and Karen L Goa. Miglustat. *Drugs*, 63:2427–2434, 2003.

[8] Astrid Nehlig. Are we dependent upon coffee and caffeine? a review on human and animal data. *Neuroscience & Biobehavioral Reviews*, 23(4):563–576, 1999.

[9] Astrid Nehlig, Jean-Luc Daval, and Gérard Debry. Caffeine and the central nervous system: mechanisms of action, biochemical, metabolic and psychostimulant effects. *Brain research reviews*, 17(2):139–170, 1992.

[10] Brian O'Donnell, Hannah Meissner, and Vikas Gupta. Dronabinol. 2020.

[11] Richard MJ Palmer, AG Ferrige, and Salvador Moncada. Nitric oxide release accounts for the biological activity of endothelium-derived relaxing factor. *Nature*, 327(6122):524–526, 1987.

[12] Donald S Robinson, Alexander Nies, C Lewis Ravaris, John O Ives, and Diantha Bartlett. Clinical pharmacology of phenelzine. *Archives of General Psychiatry*, 35(5):629–635, 1978.

[13] Erin M Rock and Linda A Parker. Cannabinoids as potential treatment for chemotherapy-induced nausea and vomiting. *Frontiers in pharmacology*, 7:207829, 2016.

[14] Abhijit Trailokya. Acenocoumarol in thromoembolic disorders. *Cardiovasc Pharm Open Access*, 4(4):1–4, 2015.

[15] Mike Ufer. Comparative pharmacokinetics of vitamin k antagonists: warfarin, phenprocoumon and acenocoumarol. *Clinical pharmacokinetics*, 44:1227–1246, 2005.
